# A low dislocation rate after revision total hip arthroplasty performed through the anterior approach

**DOI:** 10.1186/s42836-022-00159-y

**Published:** 2023-01-05

**Authors:** Edward Kahhaleh, Tatiana Charles, Xavier Collard, Marc Jayankura

**Affiliations:** 1 Department of Orthopaedic Surgery and Traumatology, C.U.B Erasme, Route de Lennik 808, 1070 Brussels, Belgium; 2 Department of Orthopaedic Surgery and Traumatology, C.H.U Ambroise Paré, Boulevard John Fitzgerald Kennedy 2, 7000 Mons, Belgium

**Keywords:** Revision total hip arthroplasty, Direct anterior approach, Dislocation, Infection, Oxford hip score, Aseptic loosening

## Abstract

**Background:**

Dislocation is a major complication in revision total hip arthroplasties. This study aimed to evaluate the dislocation rate, complications, and functional scores of revision total hip arthroplasty performed through the direct anterior approach.

**Methods:**

Between January 2014 and March 2020, 84 patients undergoing revision total hip arthroplasty were retrospectively reviewed. All operations were performed through the direct anterior approach. At the final follow-up, incidences of dislocation, reoperation, acute deep infections, periprosthetic fractures and psoas impingement were assessed. The median postoperative Oxford Hip Score was also calculated.

**Results:**

At revision surgery, the mean age was 66 ± 12 years (range, 28–91). During an average follow-up of 4.2 ± 1.2 years, reoperation rate for major complications in the non-infected revisions was 15% (*n* = 11), including five acute deep infections (7%), four periprosthetic fractures (5%), one dislocation and one psoas impingement (1%). The median postoperative Oxford Hip Score was 39 (interquartile range = 14).

**Conclusion:**

In our series, revision total hip arthroplasty through direct anterior approach was associated with a very low dislocation rate, acceptable complication rates and good functional results. Our results suggest that this procedure is safe and reliable.

**Trial registration:**

Ethical approval for this study was obtained, before enrollment of the first participant, by CUB Erasme's research ethics committee (P2020/323) and C.H.U Ambroise Paré's research ethics committee.

## Background

Total hip arthroplasty (THA) is a successful and cost-effective surgery that increases quality of life in patients suffering from hip osteoarthritis [1, 2]. An increase in life expectancy and in the number of procedures performed in younger and more active patients has led to an elevated risk of revision total hip arthroplasty (rTHA). In Germany, Klug *et* *al*. projected that, in the coming decades, a 62% increase in the incidence of THA will be accompanied by a 40% rise in revision total hip arthroplasty [3]. In the United States of America, population-based estimates have projected rTHA incidence to grow 137% between 2005 and 2030, and a recent review of registries observed an actual 43–70% increase in incidence of rTHA from 2014 to 2030 [4, 5]. Currently, over 50,000 rTHA are performed annually in the United States with a significant economic impact. The average cost of an rTHA in United States hospitals is around $50,000 [6].

Dislocation remains one of the most common complications following rTHA, with incidence rates standing between 4 and 30%. In a systematic review, Guo *et al*. reported a 9% accumulated incidence of dislocation, which usually needs another hospitalization and sometimes re-revision [7]. Dislocation following the posterior approach (PA) remains an issue, and raises revision rates [8]. Since soft tissue is preserved in the direct anterior approach (DAA), we believe that rTHA through the DAA may confer an advantage to the patients.

The purpose of this retrospective study was to show that dislocation rates are low in rTHA through the DAA. Moreover, we aimed to evaluate complication rates and functional results.

## Methods

This non-comparative retrospective observational cohort study was conducted in two Belgian hospitals after being approved by their respective ethical committees. Procedures were performed by two different orthopedic surgeons (C.X., J.M.), experienced in THA through the DAA (> 100 THAs/year) as well as hip revision surgery. Informed consent for intervention was obtained from all patients, after being informed of complications, benefits as well as postoperative rehabilitation protocol of the surgery.

We analyzed prospectively collected data of all patients who underwent rTHA through the DAA in both institutions between January 2014 and March 2020. The inclusion criteria were patients above 18 years old who underwent rTHA due to any cause, regardless of the index approach. Exclusion criteria were re-revision surgery, revisions in the presence of posterior column or wall fractures, posterior hardware removal because these revisions were done through the posterior approach. All rTHAs were done after primary hip arthroplasty. A revision procedure was defined as one requiring change of one or more of the fixed or mobile components. Revisions for infection were only reviewed for dislocation, infection recurrence and clinical score because the group was not homogenous as it included one- or two-stage revisions with different follow-ups that might produce bias.

Patients were divided into four groups according to the surgical procedures: revision of the acetabular cup alone (*n* = 27), revision of both components (*n* = 23), revision of the femoral stem alone (*n* = 20) or mobile component exchange (*n* = 4).

### Postoperative assessment

All surgical and medical reports were independently reviewed by the principal investigator (KE) to note patient and surgical, clinical evolution, follow-up time and complications. In the case of readmission, the causes of re-operation or re-revision were also recorded. The principal investigator made a phone call to the patient to calculate the 48-point inverted Oxford hip score (OHS). The maximal score referred to the best clinical results. [9]. The score was validated by prospective studies and was reliable in a clinical setting [10].

### Statistical analysis

Statistical testing was performed with the GraphPad Prism version 6.00. Kruskal-Wallis test was used to compare OHS between groups and cause-specific median OHS between subgroups. Significance was set at *P* < 0.05.

### Surgical technique

After ruling out infection, we templated the implants in terms of acetabular and/or femoral bone loss. Preoperatively, two surgeons used carefully-calibrated X-ray templating for all patients. By defining the center of rotation of the hip and the level of the stem positioning, and by calculating the implants' size and femoral offset, we minimized the error on limb length and offset restoration. Under general anaesthesia protocol specific to each institution, the patient lied supine, with a well-padded perineal support. The foot of the affected limb was secured in a boot with the patella in the neutral position. Tranexamic acid was administered to patients, with dosage tailored to their body weight. After surgical site disinfection, the operative field was covered by adhesive transparent drapes from the inferior costal margin to the ipsilateral knee, which allowed for distal or proximal extension, if needed, and checking of limb movement by surgeons. If a prior DAA incision was present, the same incision was used and care was taken to identify the intermuscular plane, as excessive scar tissue might be present. In the case of a posterior approach of the index surgery, the interval was blank, and planes were easy to access. The hip joint was exposed through the Hueter interval. Care was exercised to laterally incise the aponeurosis of the tensor fascia latae to prevent lateral femoral cutaneous nerve injuries. Gentle dissection with scissor tips was important to avoid muscle damage that could influence postoperative recovery. The thick periacetabular fibrosis was carefully and extensively resected and a V-shaped capsulotomy was made by sharply cutting it along the lateral border of the iliocapsularis from the acetabulum to the inferior insertion and then the anterior intertrochanteric line was followed just above the superior insertion of the vastus lateralis. At that point, we applied slight traction to the affected limb and disimpacted the femoral head with the use of a bone graft impactor. This allowed for easier hip dislocation.

For acetabular revision, the standard approach was used without proximal extension or release in our series. The femoral stem was retracted laterally and placed in a soft tissue pocket postero-lateral to the acetabulum. The hip was slightly flexed and a blunt section of the tendinous indirect head of the rectus femoris facilitated the exposure of the acetabulum. We placed sharp retractors at 90 degrees to achieve better exposure. The cup was removed gently using an angulated removal system (company-specific). We used curved osteotomes to free the uncemeted cups from remaining bone bridges. As for cemented cups, any persistent cement adherent to the bone was gently removed with specific instrumentation. After inspection, contained defects were filled with corticocancellous allograft bone morsels, if necessary. For larger defects, non-cemented reconstruction cages were inserted with impaction grafting. Kerboull-type cages were used for severe acetabular defects and cups were cemented into the cage.

The most important factor for femoral exposure was proper elevation and external rotation by sequentially releasing the iliofemoral ligament, pubofemoral ligament, the posterior capsule and, if needed, the short external rotators, as a last resort. The use of the orthopedic extension table with the gel bump, in our opinion, offered a highly appreciated advantage, by lowering to the ground and adducting the limb. Hence, a direct vision down the femur helped to safely extract the femoral stem [11]. No femur osteotomy for femoral stem removal was necessary for our series. To achieve maximal femoral exposure, we distally extended the incision in 3 cases in order to fix the fractures with cerclage wires, plate or both, after femoral stem exchange. Thorough washing and careful hemostasis preceded wound closure. One surgeon closed the capsule, while the other apposed it against the femoral neck. The aponeurosis of the tensor of fascia latae was closed with a running suture, with care taken not to damage the lateral femoral cutaneous nerve, which runs in the upper part of the aponeurosis. Surgical site was closed using a running intradermal suture with additional biological glue. An occlusive dressing was applied to minimize the risk of surgical wound infection. All patients, except those receiving revision for fracture and three patients with intraoperative fractures, were allowed to bear full weight from the first day after surgery. No postoperative dislocation precautions were instituted.

## Results

### Demographic details

Between January 2014 and March 2020, 84 rTHA through the DAA with complete medical records were performed in both institutions. Patients' characteristics, comorbidities that were likely to influence complications are shown in Table 1. Most of the cohort were overweight (70%), with more regular drinkers (29%) than smokers (19%). Aware of the strong association between active smoking and infections in revision surgery, smokers were advised to quit. Age at revision surgery was 66 ± 12 years (range, 28–91).

**Table 1:** Demographic details of our series

**Demographic details**	**Value**^a^	**Range or Ratio**^a^
**Number of Procedures**	84	
**Age at Revision Surgery (years)**	66 ± 12	(range, 28–91)
**Age at Index Surgery (years)**	58 ± 13	(range, 28–84)
**Time Between Index and Revision Surgery (years)**	8 ± 8	(range, 0.01–36)
**Gender**		
** Male (***n***)**	44	(52%)
** Female (***n***)**	40	(48%)
**Average BMI (Kg/m**^2^**)**	28 ± 5	(range, 17–40)
** Underweight < 18.5 (***n***)**	1	(1%)
** Normal weight 18.5–24.9 (***n***)**	25	(30%)
** Overweight 25.0–29.9 (***n***)**	29	(35%)
** Obese > 30 (***n***)**	29	(35%)
**ASA Score**		
** I (***n***)**	7	(8%)
** II (***n***)**	44	(52%)
** III (***n***)**	30	(36%)
** IV (***n***)**	3	(4%)
**Active Tobacco Users (***n***)**	16	(19%)
** Pack-Year (***n***)**	17 ± 12	
**Daily Alcohol Users (***n***)**	24	(29%)
** Daily Units (**n/day**)**	2 ± 2	

^a^Values are given as numbers and percentages, or average values with standard deviation and ranges

### Surgical data

OF all index surgeries, 54% (*n* = 45) were done through a direct anterior approach, 45% (*n* = 38) through a posterior approach and one trochanteric osteotomy. The most common cause for revision was aseptic loosening (44%, *n* = 37) of one or both component(s), followed by revision for infection (12%, *n* = 10). Five revisions for infection were performed using a one-stage operation and five using a two-stage procedure. Aseptic lymphocytic vasculitis-associated lesions related to metal-on-metal index bearing surfaces led to a rate of 12% (*n* = 10) of revisions, and periprosthetic femur fracture to a rate of 10% (*n* = 8). In the eight cases receiving revision for femoral fracture, the distal extension was necessary in three for better exposure of the femoral diaphysis. No proximal extension was necessary. These findings are summarized in Table 2.

**Table 2:** Summary of the surgical data of our series

**Date types**	**Value**^a^	**Ratio**^a^
**Index Surgery Approach (***n***)**		
** DAA**	45	(54%)
** Posterior Approach (PA)**	38	(45%)
** Trochanteric osteotomy**	1	(1%)
**Cause of Revision (***n***)**		
** Aseptic cup loosening**	15	(18%)
** Aseptic femoral loosening**	15	(18%)
** Infection**	10	(12%)
** Aseptic lymphocyte-dominant vasculitis-associated lesion (ALVAL)**	10	(12%)
** Periprosthetic femur fracture**	8	(10%)
** Total aseptic loosening**	7	(8%)
** Recurrent instability (> 1 dislocation)**	6	(7%)
** Acetabular fracture**	5	(6%)
** Psoas impingement**	2	(2%)
** Limb length discrepancy**	2	(2%)
** Alumine fracture**	2	(2%)
** Pain & stiffness**	2	(2%)
** Revision Type (***n***)**		
** Acetabulum alone**	27	(37%)
** Acetabulum and femur**	23	(31%)
** Femur alone**	20	(27%)
** Mobile components**	4	(5%)
** Length of Follow-up (years)**	4.2 ± 1.2	(range, 2.2–8.3)

^a^Values are given as numbers and percentages, or average value with standard deviation and ranges

### Complications

Table 3 details the complications occurring in different subgroups. Because the revision for the infection group was not homogenous as it included one- or two-stage revision with a different postoperative follow-up that might introduce bias, only dislocation or infection recurrence was analyzed. Out of 74 revised hips, 15% (*n* = 11) had major complications that required reoperation. All five deep infections were acute and were treated with debridement, adapted antibiotics and implant retention. All infections were eliminated, and no further complications occurred during group-specific follow-up of 2.8 years (range, 1.5–3.8), including an antibiotic-free window. Psoas impingement was treated with the endoscopic release, whereas fractures were treated with osteosynthesis alone. The only posterior dislocation occurred in the patient who had a trochanteric osteotomy as index surgery and a perioperative greater trochanter fracture during revision surgery for aseptic loosening of both components. The reduction was performed with external maneuvers followed by screw osteosynthesis of the trochanter and no recurrent dislocation occurred during follow-up. No implant revision was performed during the follow-up.

**Table 3:** Summary of complications

	**Acetabular Cup Alone**	**Acetabular Cup & Femur**	**Femur Alone**	**Mobile Components**	**Total**
	*n* = 27	*n* = 23	*n* = 20	*n* = 4	*n* = 74
**Complications that required reoperation (*n* = 11–15%)**					
**Deep Infection**	0	1	3	1	5 (7%)
**Postoperative** **Periprosthetic Fracture**	0	2	2	0	4 (5%)
**Dislocation**	0	1	0	0	1 (1%)
**Psoas Impingement**	0	0	0	1	1 (1%)
**Non-operative complications (*n* = 9–12%)**					
**Surgical Site Hematoma**	2	0	2	1	5 (7%)
**Deep Venous Thrombosis**	1	0	0	0	1 (1%)
**Surgical Wound Infection**	0	0	1	0	1 (1%)
**Wound Dehiscence**	0	1	0	0	1 (1%)
**Death From Medical** **Complications**	0	1	0	0	1 (1%)
**Total**	3	6	8	3	20 (27%)

### Patient-reported outcome measures

During an follow-up lasting 4.2 years on average (range, 2.2–8.3), the Oxford Hip Score indicated good patient-reported outcomes. The median postoperative Oxford Hip Score was 39 (Interquartile range = 14). The score was significantly higher for revision in non-infected groups compared to revision for infection, whereas no significant difference was found when comparison was made among non-infected groups. Details are shown in Table 4. On the other hand, Table 5 compares the median OHS between different subgroups. All groups showed significantly higher OHS compared to infection.

**Table 4:** Oxford Hip Scores by subgroup

	**Acetabular Cup Alone**^a^	**Acetabular Cup & Femur**^a^	**Femur Alone**^a^	**Mobile Components**	**Infection**	**Total**
**Responders/Total**	16/27	18/23	17/20	4/4	7/10	62/84
**Score 0–19**	0	0	2	1	2	5
**Score 20–29**	3	2	1	1	3	10
**Score 30–39**	6	4	3	1	2	16
**Score 40–48**	7	12	11	1	0	31
Mean Score	38 ± 7	40 ± 7	38 ± 10	31 ± 10	25 ± 7	37 ± 9

^a^Denotes significantly higher OHS compared to revision for infection group

**Table 5:** Oxford Hip Score for cause-specific revision

	**Periprosthetic Fracture**^a^	**Aseptic Loosening**^a^	**Dislocation**^a^	**Infection**
**Responders/Total**	12/13	34/37	5/6	7/10
**Median**	43	40	40	26
**Interquartile range**	9	12	2	10

^a^Denotes significantly higher OHS compared to revision for infection group

## Discussion

This study demonstrated a very low dislocation rate with rTHA performed via the DDA. Only dislocation occurred in the patients receiving a trochanteric osteotomy as index surgery. We believe that this complication happened because of insufficient repair of the abductor mechanism that was damaged by fracture, hence unbalancing the soft-tissue tension. Damage to the abductor mechanism is strongly related to dislocation rates [12]. The intermuscular DAA is abductor-sparing and reduces trauma to the soft tissues, thus making it more attractive in the setting of multiple revision surgery, specifically after another primary approach. What is even more interesting is that, by using the DAA in patients receiving posterior index surgery, we preserved the posterior neocapsule, thus avoiding eliminating this additional stability factor. As in our series, 46% of index surgeries (*n* = 39) were PA or trochanteric osteotomy approach, which reinforces our belief that revision through the DAA may offer protection from dislocations. Regarding dislocations after rTHA through posterior approach, higher rates were reported in the literature. If the posterior capsule is not repaired, which is mostly the case because of extensive capsular release, dislocation rates could reach up to 10% [13, 14], higher than rates reported in rTHA through the DAA. Thaler *et al*. reported a 7% dislocation rate in their series of 165 femoral revisions and 3% in another one of 64 acetabular revisions through the DAA [15, 16]. Prodinger *et al*. reported 3% dislocations in their prospective series of 61 acetabular revisions through the DAA [17]. Other reports described dislocation rates between 0 and 5% [11, 18–20]. Moreover, dislocation is a significant complication leading to another hospitalization with a high risk for re-revision. Yu *et al*. found that instability, as an indication for re-revision THA, was a statistically significant (*P* = 0.038) indicator of re-revision failure, with a relative risk of 1.9 (1.0–3.4) [21]. The 2020 Australian Orthopedic Association national joint replacement registry annual report described instability as the major diagnosis for a second revision. Regarding revision for infection, we did not report any dislocation of either the spacer or the definitive implants. The studies that compared the outcomes between the anterior and the posterior approaches for rTHA were scanty. Kurkis *et* *al*. have found a decreased dislocation rate of two percent compared to 13% in the PA group (*P* = 0.002) and a significantly increased risk of wound complications in the DAA cohort (7% *vs.* 0.5%), and the findings remain valid after multivariate regression analysis. In addition, a trend towards more overall 90-day complications was seen in the PA group (OR 1.71) [22]. We believe, by externally rotating and extending the hip, abductor muscle insertions on the greater trochanter are excluded from the surgical field. It may help avoid damaging them. To our knowledge, no study evaluated the muscle damage patterns after rTHA. In another series, Baba *et al*. found significantly less blood loss and total complications in acetabular revisions through the DAA compared to the PA [18]. Our study reported one wound dehiscence and one surgical wound infection, which yielded a total rate of 3% of wound complications that was in line with the rates of 1% and 12% previously reported in primary THA [23–25]. As these complications happened in two obese patients, we strongly believe that care must be taken when rTHA is done in this patient population. Preoperative aqueous chlorhexidine gluconate (4%) shows, the day before the operation, appropriate antibiotic prophylaxis before incision and daily wound care are essential to reduce this risk [26–28].

In addition, this study also demonstrated a good postoperative function in terms of the Oxford Hip Score. The use of patient-reported outcome measures (PROMs) to evaluate the clinical effect of procedures helps clinicians gain unique insight into the patients' actual and perceived physical benefits of rTHA. Our findings are in line with recent reports about functional outcomes following rTHA [29, 30]. Poor PROMs were associated with revision for infection, periprosthetic fracture or dislocation. Revision for aseptic loosening scored better on functional scores compared to revision for fracture, infection or dislocation [30, 31]. In our series, OHS functional scores in the setting of revision for infection were significantly lower compared to OHS in the setting of periprosthetic fracture, aseptic loosening and dislocation. Median comparisons between these three last groups did not yield any significant difference.

The deep infection rate in our series was comparable with published infection rates after rTHA through different approaches, ranging from 1 to 8% [22, 32]. In the setting of our study, we cannot extrapolate any conclusion about the superiority of the DAA to other approaches with respect to deep infection rates. On the other hand, in the United States Medicare population, infection is shown to be the most common complication after revision surgeries, with a total rate of 17% in 3555 revised hips between 1998 and 2011 [33]. When comparing revisions through the DAA and the PA, literature revealed no difference in the deep infection rate [18, 22]. Revision surgery is associated with longer operative time, an independent risk factor for surgical site infection and a high infection rate [34].

Regarding the risk of intraoperative fracture through the DAA, we noticed two fractures of greater trochanter tips with posterior retractor placement and one acetabular fossa fracture during cup impaction. No osteosynthesis material was used. They were treated conservatively with a protected weight-bearing protocol. These numbers are in concordance with the result reported by Thaler *et al*. who described 4 intraoperative femoral fractures (2%) in his series of 165 femoral revisions through the DAA [15]. These numbers suggest that revision through the DAA may have a low risk of intraoperative femoral fracture.

In some cases, issues like acute acetabular fractures, anterior defects or acetabular protrusion need to be addressed. With the patient assuming supine position and draped from chest to knee, all proximal and distal extensions are possible. The Stoppa approach, as well as the Levine extension of the Smith-Peterson approach, provide excellent exposure of the anterior column and could be used to treat the above-mentioned issues [35]. If needed, the distal extension provides direct visualization of the whole femur for fracture fixation, extended trochanter osteotomy or trans-femoral Wagner osteotomy for well-fixed stems [36]. Femoral retroversion makes intraoperative dislocation more challenging and acetabular exposure less easy. Moreover, other relative contraindications for rTHA through the DAA might be overweight patients with excessive skinfolds, multiple lateral incisions and the need to access the posterior column for osteosynthesis or hardware removal.

The present study has limitations due to its retrospective design and lack of a control group. Although the patient populations are comparable between the two hospitals situated in a 65-km radius where the two surgeons used the same technique, grouping bias was observed. Moreover, anesthesia and rehabilitation protocols differed from one institution to the other, introducing confounding bias that has to be taken into consideration. Furthermore, the OHS was collected by the principal investigator and not filled in by the patient, which introduced a bias and, for some patients, it was collected a year after their operations and even longer with others. The score is also sensitive to the activity level of the patient as well as the use of pain medications. We could not compare the score to a preoperative one because none was collected. Finally, when analyzing the indications throughout time, we observed that surgeons started revision through the DAA with relatively straightforward indications (acetabular cup revision) and complexity increased with time (revision of both components). This added a selection bias and showed the limitations of such an approach regarding its steep learning curve.

## Conclusion

The rTHA is increasingly performed due to a higher life expectancy. rTHA via a direct anterior approach has the potential clinical benefit of very low dislocation rates, acceptable complication rates and good functional results. We strongly feel the need for more complete and larger comparative national registry studies to provide clear evidence regarding indications, advantages and drawbacks of rTHA through the DAA compared to other approaches.

## Data Availability

The datasets generated and/or analyzed during the current study are not publicly available due to patient privacy, but are available from the corresponding author on reasonable request.

## Abbreviations

DAA: Direct anterior approach
OHS: Oxford hip score
PA: Posterior approach
rTHA: Revision total hip arthroplasty
THA: Total hip arthroplasty
